# Evaluation of the disease course of Italian children with juvenile idiopathic arthritis treated with etanercept: preliminary results in 772 patients

**DOI:** 10.1186/1546-0096-12-S1-P130

**Published:** 2014-09-17

**Authors:** Sara Verazza, Alessandro Consolaro, Cristina Robbiano, Antonella Insalaco, Rolando Cimaz, Fabrizia Corona, Giovanni Conti, Loredana Lepore, Alma Nunzia Olivieri, Donato Rigante, Francesco La Torre, Luciana Breda, Adele Civino, Gianfranco D'Angelo, Patrizia Barone, Rita Consolini, Romina Gallizzi, Maria Cristina Maggio, Maria Giannina Alpigiani, Alberto Martini, Angelo Ravelli

**Affiliations:** 1 Istituto G Gaslini, Genova, Italy; 2 Ospedale Bambin Gesù, Roma, Italy; 3 Az.Ospedaliera-Universitaria Meyer, Firenze, Italy; 4 IRCCS Ca' Granda-Ospedale Maggiore Policlinico, Milano, Italy; 5 Policlinico di Messina, Messina, Italy; 6 IRCCS Burlo Garofalo, Trieste, Italy; 7 Seconda Università degli Studi di Napoli, Napoli, Italy; 8 Policlinico Gemelli, Roma, Italy; 9 Ospedale Perrino, Brindisi, Italy; 10 Policlinico-Università di Chieti, Chieti, Italy; 11 Az. Ospedaliera di Tricase, Tricase, Italy; 12 Az. Ospedaliera delle Marche, Ancona, Italy; 13 Policlinico-Università di Catania, Catania, Italy; 14 Ospedale Santa Chiara,Università di Pisa, Pisa, Italy; 15 Università di Messina, Messina, Italy; 16 Ospedale dei Bambini, Palermo, Italy; 17 Università di Genova, Genova, Italy

## Introduction

The advent of biologic medications has considerably increased the potential for treatment benefit in juvenile idiopathic arthritis (JIA), with clinical remission being now achievable in a substantial proportion of patients.

## Objectives

To evaluate the outcome of etanercept (ETN) therapy in Italian children with JIA.

## Methods

This is a multicenter, observational study that includes all children with JIA who were given ETN at Italian pediatric rheumatology centers after January 2000. Patients were classified in 2 groups: Group 1: patients who were no longer taking ETN at study start; Group 2: patients who were still receiving ETN at study start. Patients in Group 1 underwent only retrospective assessments, whereas patients in Group 2 underwent both retrospective and cross-sectional assessments. The primary outcome of the study were reasons for ETN discontinuation in patients in Group 1, and achievement of the states of inactive disease (ID), minimal disease activity (MDA) and parent- and child-acceptable symptom state (PASS, CASS) in patients in Group 2. The above states were assessed through both formal definitions and JADAS cutoffs. The secondary outcome was the evaluation of frequency and characteristics of ETN-related side effects.

## Results

Twenty-five centers were asked to make a census of all patients followed at the center who met the inclusion criteria for Group 1 or Group 2. A total of 1230 patients were included in the census. Of these patients, 624 were still receiving ETN (Group 2), whereas 606 had discontinued ETN (Group 1). So far, the data of 772 patients (448 in Group 1 and 324 in Group 2) have been collected. Among the 448 patients in Group 1, reasons for ETN discontinuation included disease remission (57.1%), lack of efficacy (25.5%), and side effects (14.9%). The results of assessment of disease state through formal definitions in 305 children of the 324 children in Group 2 who had already undergone the cross-sectional evaluation were the following: ID 42.2%, MDA 63.8%, PASS 80.9%, CASS 76.7%. The percentages of patients who reached the same disease states assessed through JADAS cutoffs were: ID 45.7%, MDA 62.5%, PASS 71.1%, CASS 67.4%. Serious adverse events were seen in 10 of the 772 patients and included inflammatory bowel disease (4 pts), tuberculosis (1 pt), CMV hepatitis (1 pt), varicella complicated by bronchopneumonia (1 pt), bladder carcinoma (1pt), thyroid carcinoma (1 pt); 1 patient died of streptococcal sepsis.

## Conclusion

A substantial proportion of children currently receiving ETN were in the states of ID or MDA, or were satisfied with treatment outcome. More than half of the patients who had been discontinued from ETN before study start had the medication stopped because of disease remission. Serious adverse events were uncommon.

## Disclosure of interest

None declared.

